# The RCSB Protein Data Bank: views of structural biology for basic and applied research and education

**DOI:** 10.1093/nar/gku1214

**Published:** 2014-11-26

**Authors:** Peter W. Rose, Andreas Prlić, Chunxiao Bi, Wolfgang F. Bluhm, Cole H. Christie, Shuchismita Dutta, Rachel Kramer Green, David S. Goodsell, John D. Westbrook, Jesse Woo, Jasmine Young, Christine Zardecki, Helen M. Berman, Philip E. Bourne, Stephen K. Burley

**Affiliations:** 1 RCSB Protein Data Bank, San Diego Supercomputer Center, University of California San Diego, La Jolla, CA 92093, USA; 2 RCSB Protein Data Bank, Department of Chemistry and Chemical Biology and Center for Integrative Proteomics Research, Rutgers, The State University of New Jersey, Piscataway, NJ 08854, USA; 3 Department of Molecular Biology, The Scripps Research Institute, 10550 North Torrey Pines Road, La Jolla, CA 92037, USA; 4 Skaggs School of Pharmacy and Pharmaceutical Sciences, University of California San Diego, La Jolla, CA 92093, USA

## Abstract

The RCSB Protein Data Bank (RCSB PDB, http://www.rcsb.org) provides access to 3D structures of biological macromolecules and is one of the leading resources in biology and biomedicine worldwide. Our efforts over the past 2 years focused on enabling a deeper understanding of structural biology and providing new structural views of biology that support both basic and applied research and education. Herein, we describe recently introduced data annotations including integration with external biological resources, such as gene and drug databases, new visualization tools and improved support for the mobile web. We also describe access to data files, web services and open access software components to enable software developers to more effectively mine the PDB archive and related annotations. Our efforts are aimed at expanding the role of 3D structure in understanding biology and medicine.

## INTRODUCTION

The RCSB Protein Data Bank (RCSB PDB, http://www.rcsb.org) (1) develops deposition, annotation, query, analysis and visualization tools, and educational resources for the use with the PDB archive. The PDB archive is the singular global repository of the 3D atomic coordinates and related experimental data of proteins, nucleic acids and complex assemblies. It has grown to more than 104 000 entries, a 20% increase in just 2 years (2). The PDB was one of the first open access digital resources since its inception with only seven structures in 1971 (3,4). To sustain this global archive, the Worldwide PDB (wwPDB) (5,6) was formed in 2003 by three partners: RCSB PDB in the USA, PDB in Europe (http://pdbe.org) (7) and PDB in Japan (http://pdbj.org) (8). BioMagResBank (http://bmrb.wisc.edu) (9) joined the wwPDB in 2006. Together, the four wwPDB partners develop common deposition and annotation services (10), define data standards and validation criteria in collaboration with the user community (11) and task forces (12–15), develop data dictionaries (16,17) and curate data depositions according to agreed standards (18,19). Curated data files, updated weekly, are hosted on the wwPDB FTP and at wwPDB-partner mirror sites.

PDB data are loaded into the RCSB PDB relational database (20,21) and enhanced by integrating them with other biological data sources (2,22) and computed information (23) to provide a ‘Structural View of Biology’ on the RCSB PDB website. In this update, we describe characterization of protein complexes, integration of structures with protein/gene sequence and drug information. On the technical side, we report improvements to visualization, mobile support, internal software development processes, programmatic access to PDB data and annotations using web services and access to software libraries. Finally, we describe expansion of our educational offerings, PDB-101 (http://www.rcsb.org/pdb-101). Our tools and resources enable scientists to discover new relationships between sequence, structure and function, gain new insights and create new biological or biochemical hypotheses using atomic level information. Representation of structures in the context of biology and medicine and related educational resources are internet-accessible tools for high school, undergraduate and graduate level courses, and more recently Massive Open Online Courses.

## NEW WEB SITE FEATURES

### Characterization of protein complexes

Many proteins form homo- and hetero-oligomers to carry out their biological function(s) (24). For X-ray structures, however, only the atomic coordinates of the asymmetric unit representing the smallest portion of a crystal structure to which symmetry operations can be applied to generate the complete unit cell are deposited to the PDB. The asymmetric unit is, in many cases, not the biologically relevant form of a multimeric complex. One and occasionally multiple biological assemblies are assigned to each PDB entry based on experimental evidence or prediction of the most likely biological assembly by the program PISA (25). We characterize the stoichiometry and symmetry of biological assemblies and provide query and visualization tools to find and analyze them.

A large fraction of protein complexes are symmetric. Symmetry has played a central role in biology, as described in Goodsell and Olson's seminal paper on protein symmetry (24). To systematically characterize symmetry, pseudo-symmetry and protein stoichiometry (subunit composition) across all biological assemblies in the PDB archive, we have developed an efficient algorithm with which to characterize symmetry, extending earlier work by Levy (26). We begin by sequence clustering protein chains (BLASTClust, http://www.ncbi.nlm.nih.gov/) of biological assemblies at 95% and 30% identity. The 95% clusters include complexes with minor sequence variations that are often found in the PDB entries representing naturally occurring or engineered mutations. The 30% clusters group homologous complexes, and are used for identification of pseudo-symmetry. Then, the centroids of identical or homologous subunits are superposed to generate an initial transformation matrix. This transformation is subsequently applied to all Cα atoms to establish an initial mapping of subunits and the superposition is then repeated using all Cα atoms. Point group and symmetry axes for the complex are then determined from the transformation matrices. Helical symmetry is determined in a similar manner. Complexes are considered symmetric, if the Cα atom root-mean-square-deviation (RMSD) of the superposition is less than 7 Å. By analogy, structures are considered pseudo-symmetric, if Cα atoms in homologous subunits superpose within 7 Å RMSD. (N.B.: Only protein chains with at least 20 residues are considered in these calculations.)

Based on the sequence clustering of the biological assemblies, the PDB currently contains about 48 000 monomers, 38 000 homomers and 12 000 heteromers. Most homomers (96%) are symmetric and belong to cyclic, dihedral, tetrahedral, octahedral and icosahedral point groups, or they are helical.

**Figure 1.**
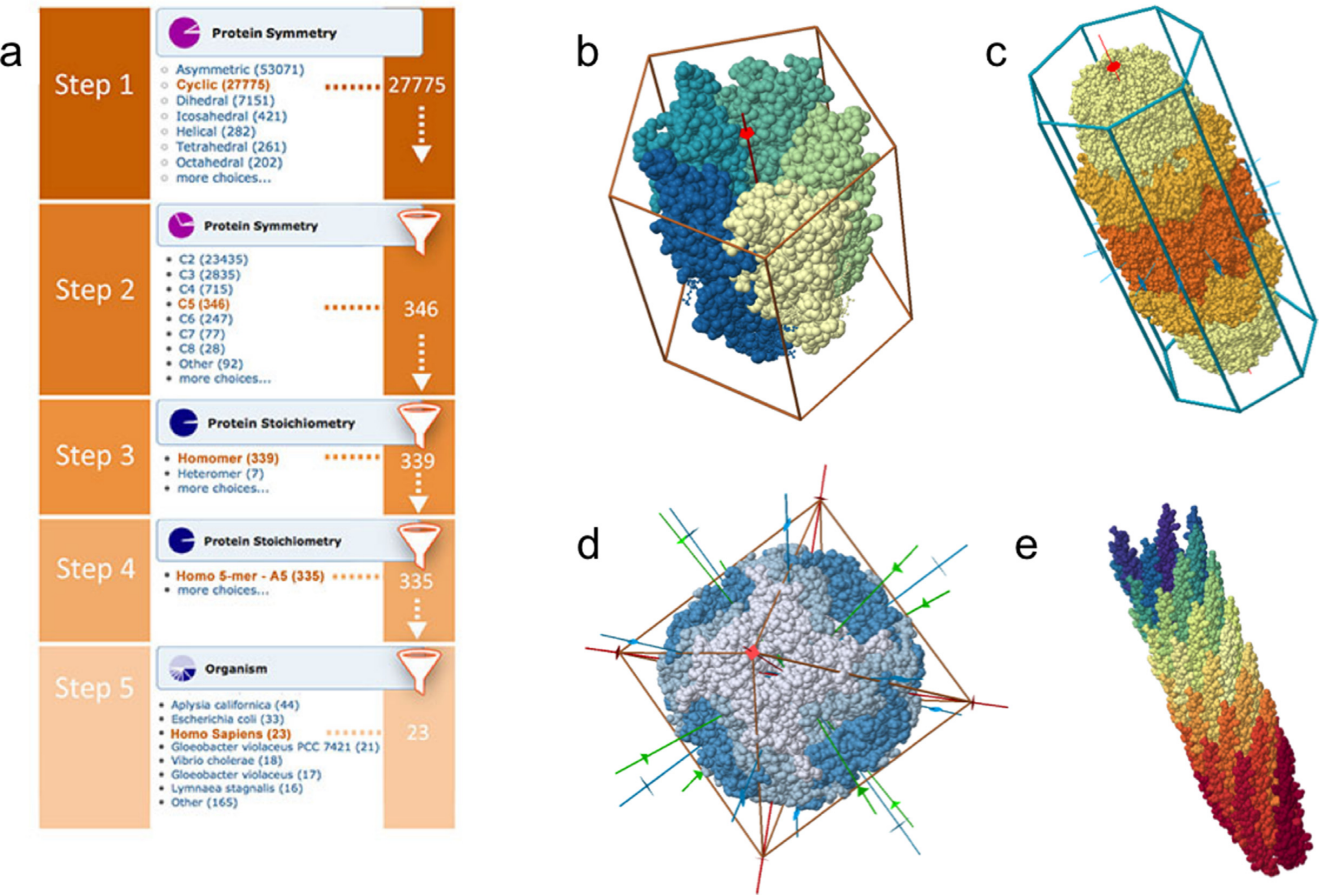
(a) Successive drill-down to find protein complexes with C5 point group that are homo-pentamers from human. The drill-down feature can be accessed from the Search panel on the home page. (b) Visualization of a symmetric protein (PDB ID 3EAM) (28) with a 5-fold axis (red), a radial color gradient to emphasize rotational symmetry and a pentagonal prism. (c) Dihedral (D7) complex (PDB ID 1YA7) (29), composed of three types of subunits (different colors). (d) Octahedral complex (PDB ID 1SHS) (30) with 4-fold (red), 3-fold (green) and 2-fold (green) axes, surrounded by an octagon. (e) Helical complex (PDB ID 1IFD) (31), displayed with a color gradient to emphasize the helical pattern of subunits.

Drill-down (Figure 1a), browse and query tools have been developed to mine these biological assembly data. Queries for stoichiometry and symmetry are available in the Advanced Search interface. The Cα atom RMSD cutoff can be specified to retrieve multimeric complexes at different levels of structural similarity. In addition, special Jmol (27) scripts have been developed to orient symmetric complexes in standard orientations along symmetry axes and display symmetry elements (Figure 1b), PDB ID 3EAM (28), PDB ID 1YA7 (29), PDB ID 1SHS (30), PDB ID 1IFD (31). Symmetry is illustrated *via* color-coding and inclusion of an appropriately shaped polyhedron enclosing the multimeric complex.

### Representation and access of small peptide-like molecules

The PDB archive also contains proteins and nucleic acids in complex with many biologically interesting molecules, some naturally derived and others of human design and manufacture. A recent wwPDB effort improved representation of a subset of the peptide-like ligand molecules using a new reference dictionary to define and standardize these so-called biologically interesting molecules in the archive (19,32). Distributed on the PDB FTP server, this **B**iologically **I**nteresting molecule **R**eference **D**ictionary (BIRD) currently contains chemical descriptions, sequence and linkage information, functional and classification information derived from both the archival entry and external resources. In the future, other types of biologically interesting molecules, such as carbohydrates, will be included in this reference dictionary.

BIRD has also enabled the RCSB PDB to offer improved searching and visualization options for these molecules. The top bar simple search and Advanced Search can query biologically interesting molecules by text/name, BIRD type (structural classification) or BIRD class (broadly defining function).

Related BIRD annotations appear on Structure Summary pages of relevant archival entries. As with other Structure Summary page features, the examples displayed (ID, name, type and class) can be used to find other PDB entries with similar characteristics. RCSB PDB 3D viewers Ligand Explorer and Protein Workshop (33) can be launched from these pages to visualize such molecules and their binding sites in the context of the full complex.

### Membrane protein annotation

Membrane proteins represent a particularly important subset of the PDB archive, because membrane proteins represent targets of about 50% of all U.S. FDA-approved drugs (34). Historically, it has proven difficult to determine their 3D structures and only in recent years has significant progress been made on these challenging targets (35,36).

In order to make the PDB entries of membrane proteins more readily accessible, we collaborate with the *mpstruc* database (S. White, Membrane Proteins of Known 3D Structure, http://blanco.biomol.uci.edu/mpstruc/). This database currently contains a classification of membrane-related proteins, encompassing ∼1500 PDB entries. The RCSB PDB implemented a computational pipeline to extend this classification to additional PDB entries, by analyzing sequence clusters recalculated weekly. If *mpstruc* annotates a PDB chain as being a membrane protein, all members of the sequence cluster containing that chain at 90% sequence identity are inferred to share the same membrane classification. Using this approach, it has been possible to extend the membrane structure classification to ∼2500 PDB entries.

The new RCSB PDB membrane structure classification can be accessed either by drilling down through the auto-suggest results at the top-bar search, by selecting the transmembrane browser from the Browse Database feature or by typing the word ‘membrane’ in the top-bar search and selecting the ‘Retrieve’ ‘Membrane Proteins’ option.

### Drugs and drug target mapping

Co-crystal structures with drugs, lead compounds and drug fragments play important roles in drug discovery. We have improved searching for drugs within the PDB using both generic and brand names by mapping ligands in the archive to molecules in DrugBank (37). A user can simply type in the name of drug into the top bar search box of our website, and if the drug exists in the PDB, the auto-suggest feature will provide a direct link to the pertinent drug ligand summary page.

#### Mapping to DrugBank

DrugBank provides a rich resource for both experimental and approved drugs, and their targets. We have established two mappings between DrugBank and the PDB archive. First, we map drugs to PDB ligands by comparing the standard InChI key (38), a unique linear string representation of a molecule, for PDB ligands to drug molecules in DrugBank. If the InChI key of the PDB ligand is identical to that of a drug molecule, then it is annotated as a stereochemical match. If only the first 14 characters of the InChI key match, which represents an encoding of the molecular connectivity, then the PDB ligand is annotated as a non-stereo match. Non-stereo matches can arise, for example, when the ligand in the PDB is a steroisomer of the drug molecule, the drug is a racemic mixture (e.g., Thalidomide, DB0141), or stereochemistry information is not available (e.g. Atorvastatin, DB01076) from DrugBank. If the drug molecule is bound to a protein, we characterize the identity between the protein sequence of the PDB entry with the primary (pharmacological) drug target annotated by DrugBank. In addition, we map drug targets in DrugBank to protein sequences in the PDB, even if the archival entry contains no bound ligand or a non-drug bound ligand.

#### Tables of drugs and drug targets

**Figure 2.**
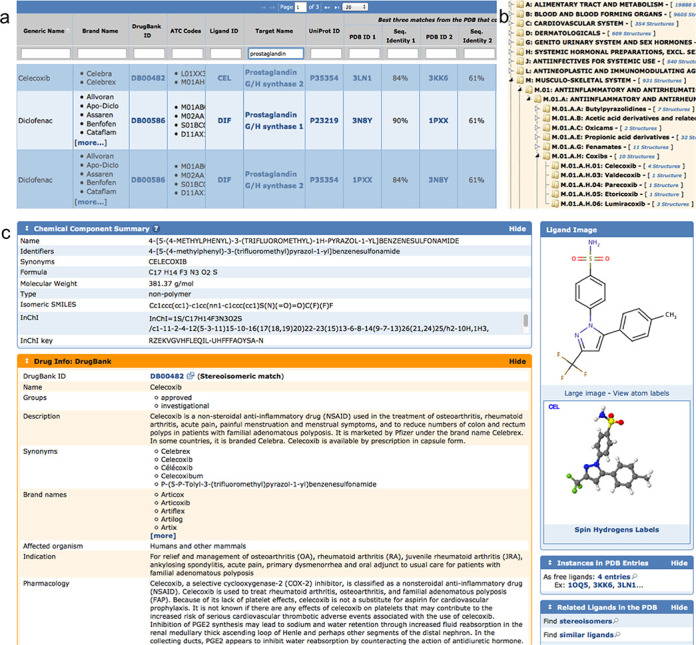
(a) Section of drug mapping table for prostaglandins with some of the hits shown for Celexocib and Diclofenac. On the right side of the table are links to related PDB entries and% sequence identity compared to the target sequence. (b) ATC tree with anti-inflammatory section expanded, showing Celecoxib and related drugs represented in the PDB. (c) Ligand summary page for Celecoxib, enhanced with DrugBank annotation (orange box). At the bottom right are links to all four entries that contain Celecoxib, plus a search link to find similar ligands.

The results of mapping DrugBank and PDB data are updated weekly and made available as tabular reports that can be searched, sorted and exported as Excel or comma-separated value files. One of the tabular reports lists drug molecules in the PDB that are bound to their primary drug target(s) or their homologs. Up to three best matching PDB IDs are listed together with the% sequence identity to the primary drug target (Figure 2a). A second table lists drugs and their primary drug targets, even if there is no drug–drug target complex in the PDB.

#### Hierarchical browsing of drug containing structures

By combining the Anatomical Therapeutic Chemical (ATC) classification annotation provided by DrugBank with the drug mapping to PDB, we have created an ‘ATC Browser’ (Figure 2b). ATC is a drug classification system developed by the Word Health Organization Collaborating Centre for Drug Statistics Methodology (http://www.whocc.no/atc_ddd_index/). Our ATC Browser allows the user to navigate the ATC hierarchy, first using the anatomical classification, followed by therapeutic classification and finally by the chemical class of the drug molecule. Each level of this hierarchy is linked to relevant structures in the PDB. For example, in Figure 2b there are 931 structures at the top level related to the muscular-skeletal system (anatomical classification), 112 structures related to anti-inflammatory and anti-rheumatic products, non-steroids (therapeutic classification) and 10 structures related to the coxibs (chemical classification), and 4 structures that contain Celecoxib. In addition, we can see that there are also structures in the PDB archive containing related drugs, including Valdecoxib, Parecoxib, Ethoricoxib and Lumiracoxib.

#### Ligand summary page annotations

Each ligand (chemical component) in the PDB has an associated ligand summary page (Figure 2c) that lists basic chemical information (18) including names, molecular weight and molecular descriptors, such as SMILES (39), InChI and InChIKey, plus a 2D chemical diagram and a 3D interactive view. Since many ligands have important pharmacological functions, we have provided additional annotation by integrating data from DrugBank. For ligands that match DrugBank entries, we display DrugBank annotation and links to the associated DrugBank drug cards (Figure 2c). DrugBank also annotates secondary targets and off-targets. By clicking on the search link, the user can use the RCSB PDB sequence search to find secondary targets and off-targets. For example, 3-phosphoinositide-dependent protein kinase 1 is listed as a secondary target for Celecoxib, and the search returns several PDB structures of this protein. Celecoxib and its derivatives are known to inhibit 3-phosphoinositide-dependent protein kinase 1, which is implicated in tumor invasion, angiogenesis and tumor progression (40). This example illustrates the utility of linking two complementary data resources to aid the user in exploring such relationships.

### Binding site visualization

**Figure 3.**
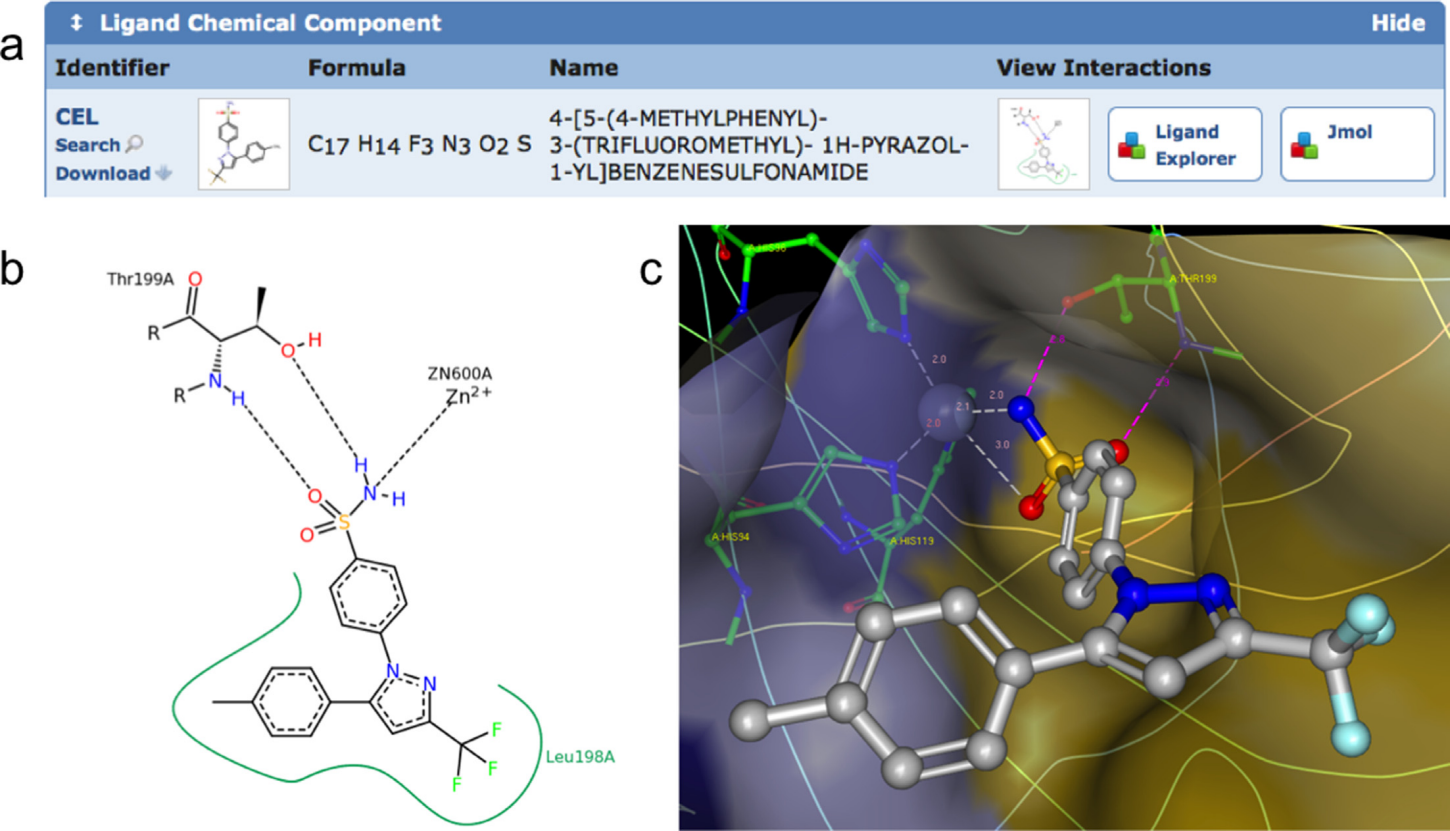
(a) Ligand Chemical Component widget on the Structure Summary page for Celecoxib bound to carbonic anhydrase II from PDB ID 1OQ5 (41). The ‘View Interactions’ section provides access to 2D and 3D ligand-binding site views. (b) 2D Ligand interaction diagram generated by PoseView (42) (dashed lines, hydrogen bonds and metal interactions; green line, hydrophobic interactions). (c) Interactive 3D visualization using Ligand Explorer (33) (ligand in stick and ball style, and some of the interacting binding site residues in green; dashed lines, hydrogen bonds and metal coordination with distance, and transparent binding site surface color-coded by hydrophobicity of the closest amino acid residue (yellow, hydrophobic; blue, hydrophilic).

**Figure 4.**
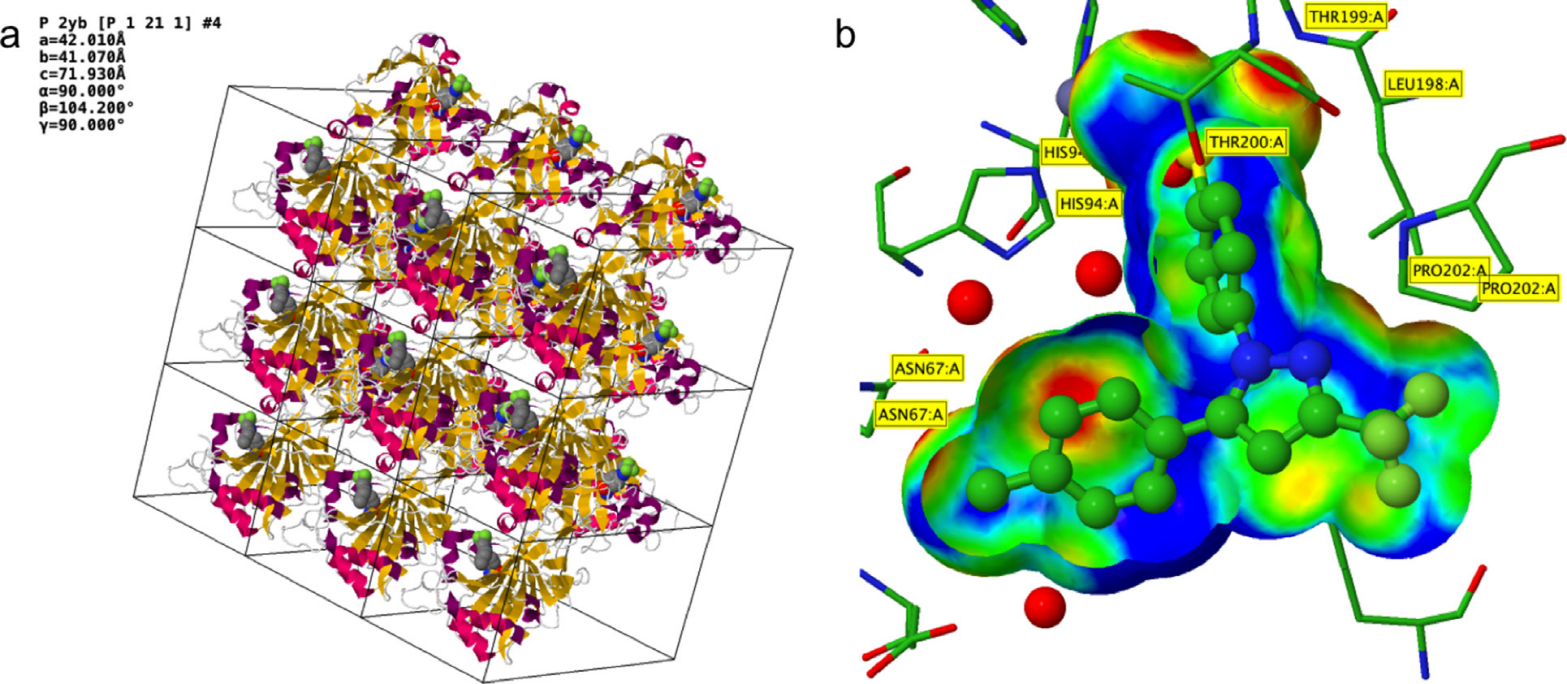
Two new custom rendering options on the Jmol /JSmol page demonstrated using PDB ID 1OQ5 (Celecoxib bound to Carbonic Anhydrase II) (41). (a) Rendering of the crystal packing (3 × 3 unit cells in the ab plane), carbonic anhydrase II (cartoon style) and Celecoxib (CPK style). (b) Jmol rendering of the Celecoxib (ball-and-stick style) in carbonic anhydrase II binding site (stick style). The trimmed ligand VDW surface is color-coded by the distance of the surface to the nearest side chain atom (short contact distances, red; optimal hydrophobic interaction distances, green; longer distances, blue). The red regions (close contacts) at the top of the surface represent hydrogen bonds and metal coordination of the sulfonamide group of Celecoxib with THR 199 and Zinc.

Co-crystal structures of drugs available in the PDB offer atomic level views of drug-macromolecule interactions. We provide visualization tools to help examine these interactions. Each PDB structure has an associated Structure Summary page. If a structure contains ligands, they are enumerated in the Ligand Chemical Component section (Figure 3a) of the Structure Summary page, PDB ID 1OQ5 (41). Ligand analysis options include a 2D ligand interaction diagram generated by PoseView (42) (Figure 3b), interactive 3D binding site views using Ligand Explorer (33) (a Java Webstart application automatically installed and updated on client machines) and Jmol applet (27) (Figure 4b). Ligand Explorer provides options to display hydrogen bonds, metal coordination, hydrophobic contacts, plus solid, mesh and dotted binding site surfaces colored for hydrophobicity.

### JavaScript applications to support mobile devices

To support growing web traffic from mobile devices, we now offer JavaScript-based applications as an alternative for unsupported Java Applets. We have replaced the Marvin Java Applet with the new JavaScript version, MarvinJS (https://www.chemaxon.com/) for chemical structure searching. This augmentation provides for smooth integration of the application with our website and works well on any device. For 3D display of ligands on the Ligand Summary pages and small proteins within the PDB-101 educational section, Jmol (Java Applet) has been replaced with its JavaScript version, JSmol (27). For the main Jmol viewer hosted on Structure Summary pages, Jmol is used if Java support is detected; otherwise, JSmol is launched.

For high-performance rendering and display of very large structures on mobile devices, we offer the RCSB PDB *Mobile* app for both iOS and Android operating systems (43).

### New 3D structure visualization features

Two new visualization features have been added to the Jmol 3D viewer offered from Structure Summary pages. A crystal packing option displays the asymmetric unit, unit cell and up to three copies of the unit cell along each of the three crystallographic axes (Figure 4a). An option to visualize ligand-binding sites in Jmol is also available (Figure 4b).

### Protein feature view

Many PDB entries do not contain atomic level data for an entire polypeptide chain. In some cases an experiment may have focused on just one domain of a multidomain protein, or it may not have been possible to determine atomic coordinates for every residue in the polypeptide chain. To clarify how PDB entries relate to the full-length protein sequence, we developed a new protein sequence viewer, the *Protein Feature View*. It combines annotations from various data sources and provides a graphical summary of any annotations that can be mapped onto the protein sequence or 3D structure.

**Figure 5.**
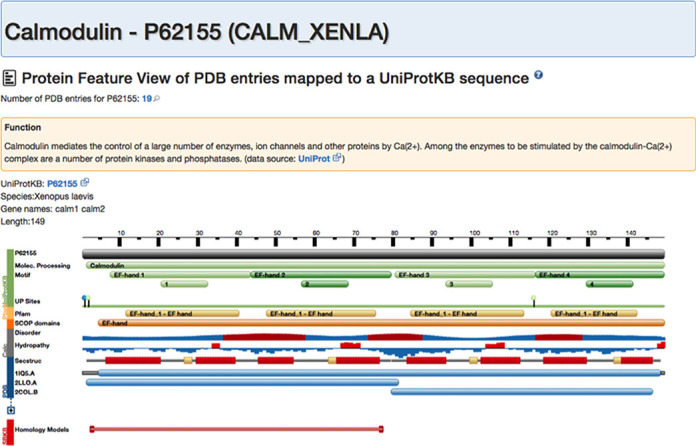
Protein feature view for Calmodulin. The main reference (gray track) is the full-length sequence from UniProt. The top of the view (green) provides a summary of important functional motifs plus UniProt sites. By moving the mouse over any of these regions, more information about this region can be viewed. This is followed by annotations from Pfam (yellow), SCOP domain annotations (orange), computationally inferred information, such as protein disorder score or hydropathy. At the bottom, in blue, are the data derived from PDB. A secondary-structure track shows helical and strand regions along the protein. In the ‘condensed’ (default) view representative protein chains are being displayed mapped to the protein sequence. To view the mapping of all available PDB chains the ‘expanded’ view can be called by pressing the ‘+’ button.

For data visualization, so-called ‘tracks’ are used (Figure 5). Color-coding on the left side of the view indicates the provenance of a track, e.g. all information coming from UniProt (44) is in green, PDB information is in blue, etc. In some cases, we are dealing with manually curated information, such as SCOP domains (45). Alternatively, the annotation has been inferred indirectly, as for Pfam domains (46), or calculated, as for hydropathy scores (47) and propensity for protein disorder score based on JRONN (P. Troshin and G. J. Barton, unpublished), a Java implementation of RONN (48).

A compressed version of the Protein Feature View is available on all Structure Summary pages, where it provides a glimpse of every protein chain within a given PDB entry (up to a maximum of 10, for performance reasons). The full-scale version of the view provides more detailed information and can be accessed from the Structure Summary or by searching for UniProt accession codes in the top bar search.

We supplemented the Protein Feature View with textual annotation provided by UniProt. If available, we also add functional context, including domain organization, oligomeric structure and catalytic activity. If references to other protein entries are identified within these textual descriptions, we provide links to navigate between relevant entries.

### Gene view

**Figure 6.**
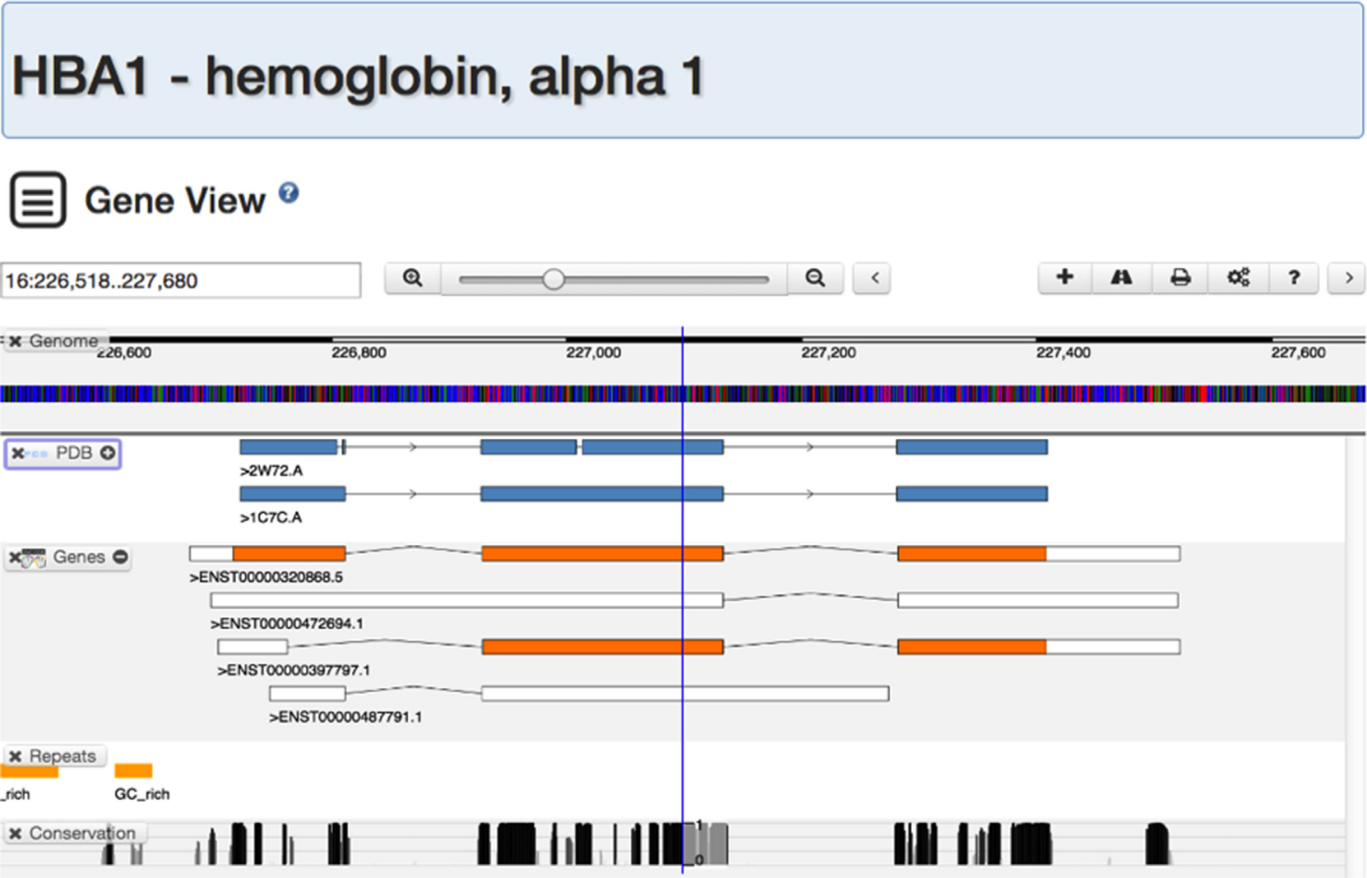
Gene view of human chromosome 16, zoomed into the region of the HBA1, hemoglobin α1 gene. The top of the view is a ruler that shows the current region along a chromosome. The blue/red/green/black colored tracks is the color-coding of the nucleotides. The PDB track shows two PDB chains: PDB ID 2W72 chain A (50), PDB ID 1C7C chain A (51) and how they align to this region on the chromosome. The Genes track shows four transcripts, one of which is the main HBA1 coding sequence. By zooming out, the human α globin gene cluster, spanning about 30 kb and including several hemoglobin genes, can be visualized.

To enable better integration between the growing corpus of genomic data and 3D information in the PDB, we implemented a pipeline that maps protein structure data onto the human genome. We can now link most human PDB entries to their corresponding genes. To visualize this mapping, we created Gene View to support browsing of the human genome with PDB data mapped onto corresponding genomic positions. These data can be correlated with other genomic annotations, such as gene structure prediction, DNA repeats or sequence conservation, across ∼50 vertebrate genomes. Our graphical view is built atop the BioDalliance genome browser (49). Users can ‘mouse’ through the region around a human gene, as shown in the Gene View (Figure 6), PDB ID 2W72 chain A (50), PDB ID 1C7C chain A (51).

Like Protein Feature View, Gene View displays information in tracks (Table 1), Genes (52), UCSC Genome Browser (53), PhastCons Score (54), which can be reordered at will by dragging the title box. The view can also be zoomed in or out. Gene View can be launched from a Structure Summary page for a human protein under ‘Molecular Description,’ or by searching for human gene names in the top-bar search.

**Table 1.** Description of Gene View tracks

Gene View track	Description
Genome	Position of the region of the genome and the nucleotide (when zoomed out as a letter code, when zoomed in as a color-coded region, guanine: black, adenine: green, cytosine: blue, thymine: red).
PDB	Region of the gene with available PDB protein chains. Clicking on the region displays more information about the mapped protein.
Genes	Annotated gene, as provided by the Gencode project (52). White boxes represent UTRs (untranslated regions) and orange boxes represent protein-coding regions. Black lines connecting boxes represent introns.
Repeats	Regions that have been annotated to contain various repeats. Data provided by UCSC Genome Browser (53).
Conservation	PhastCons conservation scores (54) derived from multiple alignments of 45 vertebrate genomes to the human genome. Data provided by UCSC Genome Browser.

Mapping of PDB entries to the human genome is also available as a table and as a single file for download (http://www.rcsb.org/pdb/browse/homo_sapiens.do).

### Structure validation information

RCSB PDB provides access to geometric validation and other information, including the Validation Reports for all X-ray structures generated by the wwPDB (55). These reports contain quality assessments for each structure and highlight specific concerns by considering the atomic coordinates, the experimental data and the goodness-of-fit between the two. Easily interpretable summary information that compares structure quality to other archival entries helps PDB users critically assess and select the most appropriate structures for their needs. In addition, a graphical summary of the quality of all polymeric chains at the residue level provides an in-depth view of quality. The Validation Reports were designed in consultation with a large group of community experts (12), and developed in the context of a larger initiative, the new wwPDB Deposition and Annotation system (10).

Validation Report ‘slider’ graphics are prominently displayed on RCSB PDB Structure Summary pages, together with a link to the PDF file containing the entire report. Metrics shown in the ‘slider’ graphic compare several important global quality indicators for this structure with those of previously deposited PDB entries. In general, an entry with all sliders in the blue areas (right-hand side) is of superior quality. In addition, links to Ramachandran plots created by MolProbity (56) are available to provide a quantitative assessment of the conformational quality of protein structures.

Access to validation information for nuclear magnetic resonance (NMR) and cryo-electron microscopy structures following the recommendations of the NMR (14) and electron microscopy (EM) (13) Task Forces is under development by the wwPDB.

### Broad promotion of a structural view of biology

RCSB PDB develops tools and resources to promote the study and exploration of PDB data, biology and biomedicine to diverse audiences, ranging from experts to users new to the molecular world. Our goal is to provide assistance wherever needed, and to streamline access for users already familiar with the archive. The What's New page (www.rcsb.org/pdb/static.do?p=general_information/whats_new.jsp) and the Help System (http://www.rcsb.org/pdb/staticHelp.do?p=help/index.html) guide users to both new and existing features of the website.

One such tool, RCSB PDB *Mobile*, fully described in (43), supports simple queries of the PDB archive, browsing of results and access to the complete catalog of *Molecule of the Month* education articles using mobile devices. RCSB PDB *Mobile* also allows interactive 3D visualization using the molecular viewer NDKmol (http://webglmol.sourceforge.jp), launched by entering a PDB ID or from search results. Images can be saved to the Camera Roll.

The initial app was released for iPhones and iPods in 2010, and now as an app for Android. It has been downloaded from the App Store and Google Play more than 25 000 times.

Our principal educational resource is the PDB-101 website (http://www.rcsb.org/pdb-101), which packages together materials and activities to support teachers and students, general audiences and non-structural biology experts looking for information about PDB data and biology/biomedicine. This website, accessible by clicking the PDB-101 logo yet separate from the main website at rcsb.org, was launched in 2011 and accounts for ∼5% of all RCSB PDB traffic. PDB-101 draws upon the archive of more than 175 *Molecule of the Month* articles to support a top-down browser that helps users find structures based on areas of biological or biomedical interest and to provide open access to high-resolution images. PDB-101 offers a variety of educational flyers and animations, such as a video and flyer that aims to answer the question *What is a Protein?* PDFs can be downloaded and used to create 3D paper models of DNA, tRNA, dengue virus, HIV capsid and green fluorescent protein. A 2014 RCSB Video Challenge inspired high school students across the United States to create and submit short videos that tell a story about HIV/AIDS at the structural level. An impressive array of science and storytelling techniques was shown in the videos submitted and available from the PDB-101 website. This competition will be repeated in the Spring of 2015.

### Access to data files and web services

Programmatic access to PDB structures and associated data and annotations is essential for bioinformatics analysis, data mining and structure-based drug discovery. The PDB offers access to data files, including atomics coordinates and primary experimental data, such as structure factor and chemical shift files (Table 2). In addition, the RCSB PDB provides sequence clusters, generated by BLASTClust, and clusters of structures determined by multiple methods (57).

**Table 2.** Data files available from the wwPDB and RCSB PDB ftp sites for download

Resource	Description	URL
PDB Archive	PDB structure data, structure factor files, NMR restraint files, NMR chemical shift files, validation reports; Chemical Component Dictionary, BIRD dictionary	ftp://ftp.wwpdb.org/
		Data mirrors:
		ftp://ftp.ebi.ac.uk/pub/databases/pdb/
		ftp://ftp.pdbj.org/pub/pdb/
PDB Archive/EM	EM maps associated with EM structures in the PDB	ftp://ftp.wwpdb.org/pub/emdb/
Sequence Clusters	Grouped PDB protein sequences clustered from 30% to 100% sequence identity	ftp://resources.rcsb.org/sequence/clusters/
Structures determined by multiple methods	Clusters of structures determined by multiple methods (57)	ftp://resources.rcsb.org/sequence/clusters/

RESTful Web Services provide customized services to run queries and fetch specific data from the RCSB PDB database (Table 3). RESTful services are platform-independent and easily accessible from any programming or scripting language. Sample code in Java, Python and Perl is available from the RESTful services page. KNIME workflow nodes to access RESTful web services are available in the KNIME workflow environment (http://tech.knime.org/book/vernalis-nodes-for-knime-trusted-extension) and were developed by Vernalis, plc. For example, the KNIME PDB Connector Node first executes an advanced search, and then creates a tabular report as output that can be further processed by other KNIME workflow nodes.

**Table 3.** Overview of RCSB PDB Web Services

Web Service	Description
Generic Search service	Run any simple, advanced, or Boolean combination of searches available from the website; Uses an XML representation to describe a query
Tabular Report service	Create custom tables of primary PDB data and external annotations for all or a specified set of PDB structures
Description services	Return descriptions of PDB entries and chemical components
Status services	Return the release status of PDB entries, the current set of release and unreleased entries, pre-released sequences of unreleased entries and obsolete entries
Chemical Structure services	Run substructure, superstructure and similarity searches for PDB ligands using a SMILES string
Annotation services	Return mapping of annotations to PDB chains. Specific services exist to retrieve UniProtKB, Pfam and GO annotations
Sequence Cluster services	Return specific sequence clusters for a given protein chain or all chains at a given level of sequence identity

A detailed description of all available services is found at http://www.rcsb.org/pdb/software/rest.do.

### Software development practices and open source software

To ensure rapid development, we have recently adopted continuous integration as the principle governing our software development activities. When RCSB PDB developers commit new source code to our code-repository, an automated build system will try to compile all code and run all JUnit-test (http://junit.org) cases that are part of the project. If everything compiles correctly a copy of the code will be deployed on a test server and a developmental version of the RCSB PDB website is made available for internal use. If things go wrong, an alert is sent to the developer(s), pointing out the problem. This approach also allows for rapid deployment of new versions of the production website. An internal command-and-control center monitors the condition of our production servers (currently there are nine servers each on the East Coast at Rutgers and the West Coast at UC San Diego). A command-and-control center enables deployment of a new build straight from the automated build environment with a few mouse clicks, thereby resulting in much faster software development life cycles.

We have also worked on making our software more modular and sharing parts of our code base with the public as open source. BioJava (47) is an open framework for rapid development of bioinformatics applications. We have contributed a significant amount of our scientific software, such as structure alignment algorithms, correct representation of protein structural data, calculation of protein symmetry, plus various other algorithms for analysis of proteins to BioJava. We use BioJava for computing additional data for the RCSB PDB website. For example, the protein disorder and hydropathy tracks of the Protein Feature View are computed using software contributed to BioJava by a Google Summer of Code student.

BioJava is hosted as an open source project on GitHub (https://github.com/biojava). The RCSB PDB GitHub repository (https://github.com/rcsb) comprises a collection of open source projects, not part of BioJava, including the RCSB PDB viewers (Ligand Explorer, Protein Workshop, Simple Viewer and Kiosk Viewer), the Symmetry and Protein Feature View projects.

## SUMMARY

The RCSB PDB has developed new annotations, tools and structural views of biology and medicine for PDB data to enable a deeper understanding of biology for both basic and applied research and education. Going forward, we will address new challenges by catering to the needs of an ever expanding user community, further improving the user experience on both the web and mobile devices, handling very large structural complexes coming from integrative structural biology and providing tools to mine the PDB archive and assess the quality of entries determined by NMR, EM and other experimental techniques.
